# Hemisection: A Boon for the Hopeless Tooth

**DOI:** 10.7759/cureus.59967

**Published:** 2024-05-09

**Authors:** Rohan R Khetan, Joyeeta Mahapatra

**Affiliations:** 1 Conservative Dentistry and Endodontics, Sharad Pawar Dental College and Hospital, Datta Meghe Institute of Higher Education and Research, Wardha, IND

**Keywords:** bicuspidization, tooth sectioning, tooth resection, endo-perio lesion, endodontic surgery, hemisection

## Abstract

If left untreated, an inflammatory periodontal disease eventually leads to attachment loss. This may have an impact on a multi-rooted tooth's bifurcation or trifurcation. The division of a tooth with two roots into two distinct parts is known as hemisection. *Hemisection* is the term used to describe the removal or separation of a two-rooted tooth's root and crown, most likely a mandibular molar. Compared to other treatment options, hemi-sectioning the affected tooth can help preserve the tooth's structure and alveolar bone. Careful selection of cases is essential for the long-term success of the procedure. In this case report, in contrast to the more common option of extracting the natural tooth, a treatment option is discussed for molars with extensive decay that threatens tooth loss. Therefore, this option should be discussed with patients when deciding on a course of treatment, and it may be a good substitute for extraction and implant therapy, particularly in cases of advanced endo-perio lesions.

## Introduction

One of dentistry's main objectives is to preserve the natural dentition. In addition to valuing their teeth, patients nowadays also say they would prefer to preserve their natural dentition rather than have them extracted whenever feasible. Patients now have the chance to preserve a functional dentition for the rest of their lives thanks to recent advancements in dentistry. Hemisection is the surgical term for extracting one root and the overhanging crown from a tooth with multiple roots. It is usually done in mandibular molars. In case selection, if a tooth is indicated for hemisection, it must first receive endodontic therapy [1]. When a root is removed selectively, it leads to a reduction in the pocket depth and an increase in the accessibility for better oral hygiene, thus controlling the plaque. This, in turn, leads to healthy bone formation. If the periodontal disease is not treated and the tissues' health is not restored timely, it could result in complete tooth loss. Therefore, rather than extracting the tooth in toto, this kind of conservative procedure helps preserve as much of the tooth structure as possible [2]. Hemisection is the sectioning of a molar after irreversible damage to one of the tooth root-which could be impacted by a carious lesion, endodontic infection, or periodontal disease that is eventually eliminated [3].

The various periodontal indications include Severe angular bone loss in teeth with multiple roots, affecting only one root, grade III or IV involvement in furcations, the adjacent teeth roots' unfavorable proximity, and dehiscence that exposes the roots severely. The various endodontic and restorative indications are a splint's abutments failing prosthetically when one of the roots of a tooth with endodontic involvement had an uninstrumentable perforation in the pulp chamber floor or pulp canal, a vertically fractured root, traumatic injury resulting in significant damage and furcation involvement or sub-gingival caries. Indications for hemisection include when a multi-rooted tooth has periodontal disease, vertical root fracture, or caries affecting just one root, a surviving root that can be accessible and treated endodontically, the surviving root should be able to support a post and core restoration structurally, the remaining root needs to be positioned so that it doesn't obstruct the fixed prosthetic restoration's insertion path. The contra-indications for hemisection include fused or poorly shaped roots, situations in which endodontic therapy cannot be performed, and a patient who doesn't comply [4]. The case report aimed to show how hemisection of tooth number 36 can be successfully managed with occlusal rehabilitation. This conservative treatment approach aimed to preserve as much of the original tooth structure as possible.

## Case presentation

A 49-year-old male patient reported to the Department of Conservative Dentistry and Endodontics, Datta Meghe Institute of Higher Education and Research, Wardha, India, with the chief complaint of pain in the lower left back region of the jaw for one month. The pain was dull, insidious in onset, throbbing in nature, and it gets aggravated on mastication of food. 

There was no significant medical history as well as dental history. On clinical examination, disto-proximal caries were seen with 36, which was tender on percussion. The provisional diagnosis was pulp necrosis with symptomatic apical periodontitis with tooth number 36. On electric pulp testing, no response was recorded with tooth number 36. The contralateral control tooth was 46.

An intraoral periapical radiograph (IOPA) revealed extensive radiolucency in the crown involving enamel, dentin, and pulp suggestive of gross carious lesion. The radiolucency extended to the radicular area of the distal root of the 36, suggestive of root caries. Periodontal ligament (PDL) space widening was observed in the apical section of the 36, suggesting apical periodontitis. The final diagnosis was pulp necrosis associated with symptomatic apical periodontitis in relation to tooth number 36.

**Figure 1:**
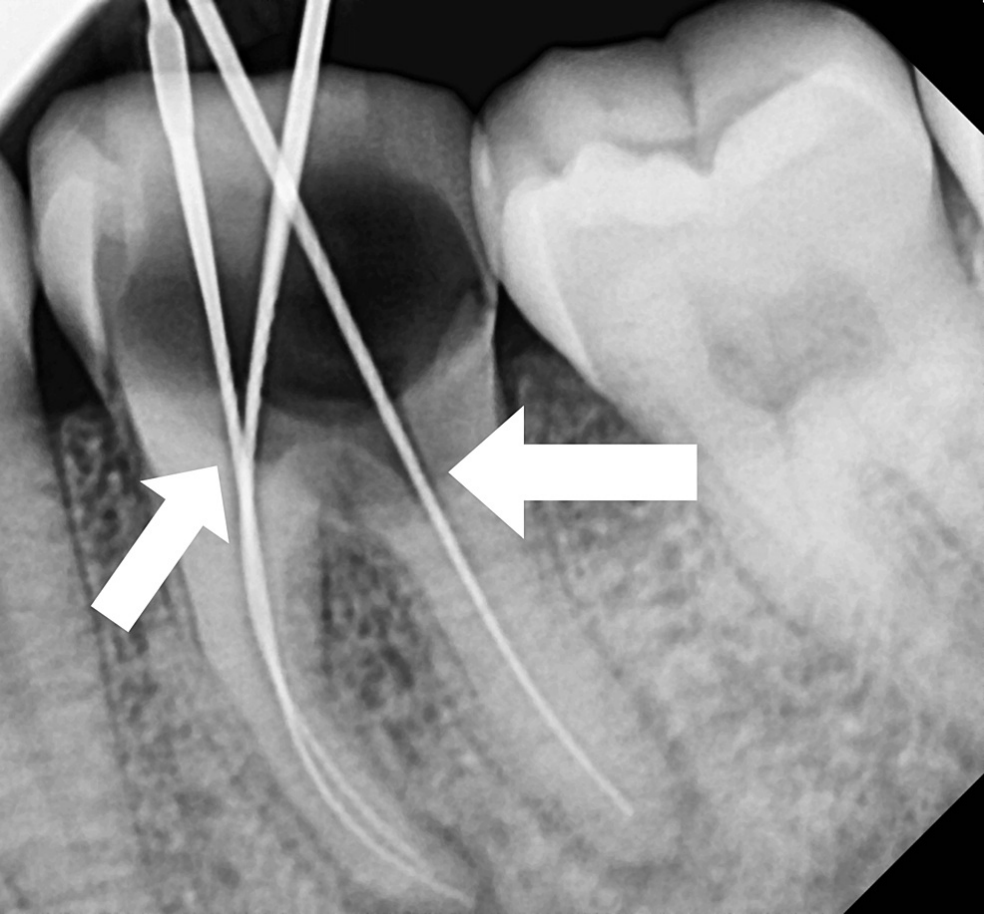
Working length of tooth number 36

The patient was informed about the treatment plan, and his written informed consent was acquired. The treatment options given to the patients were A) Extraction followed by implant, B) Extraction followed by bridge prosthesis, and C) Hemisection followed by crown prosthesis. Before starting the root canal process, oral prophylaxis was carried out. Tooth number 36 was isolated using a rubber dam (Ivoclar Optradam Plus, Liechtenstein). The access cavity was prepared using the round bur (BR-45, Mani, Japan) and the safe end bur (EX 24, Mani, Japan). Three canals were located, i.e., mesiobuccal (MB), mesiolingual (ML), and distal (D). Using a radiograph and an electronic apex locator (J Morita Root ZX Mini, Tokyo, Japan), the working length was ascertained. Length of operation with MB = 18 mm, ML = 19 mm, and D = 17 mm (Figure 1).

To remove any remaining pulp tissue and debris, the canals were irrigated with 3% sodium hypochlorite (Vishal Dentocare Pvt Ltd, Ahmedabad, Gujarat, India), followed by 0.9% normal saline. Biomechanical preparation was completed in every canal up to the F2 ProTaper rotary file(Dentsply ProTaper Gold Refills NiTi Rotary Files, Germany). The cavity is then rinsed with 2% Chlorhexidine to reduce the induction of the cytokine reaction that will further arrest the progression of the inflammation in the canal, which will lead to a decrease in the inflammation in the periapical space [5]. The Intracanal medicament dressing of calcium hydroxide (Prime RC Cal, India) was placed, followed by the application of a temporary pack (Maarc T-fill, Belgium). The patient was called back for a follow-up appointment after five days.

**Figure 2:**
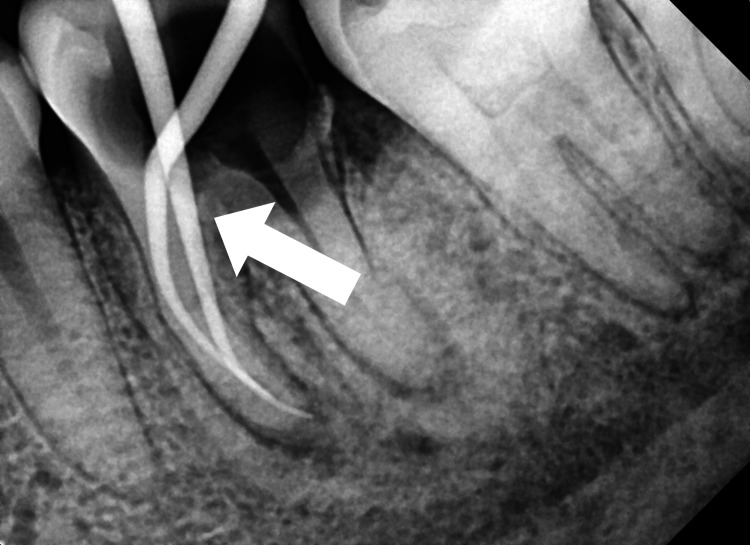
Master cone fit in the mesiobuccal and mesiolingual root canal of tooth number 36.

During the second follow-up visit, the temporary dressing was taken off, and the canal was irrigated with saline, liquid Ethylenediamine tetraacetic acid (EDTA) (Ammdent, Mohali, India), which was activated with the endoactivator (Dentsply Sirona Endodontics, Tulsa, United States) to flush out the calcium hydroxide from the canal. Master cones Gutta-percha (Dentsply Maillefer, Tulsa, Oklahoma, US) corresponding to the sizes of the last files used in the canal preparation of MB and ML are selected,, respectively, and a periapical radiograph was recorded to check for the cone fit (Figure 2).

**Figure 3:**
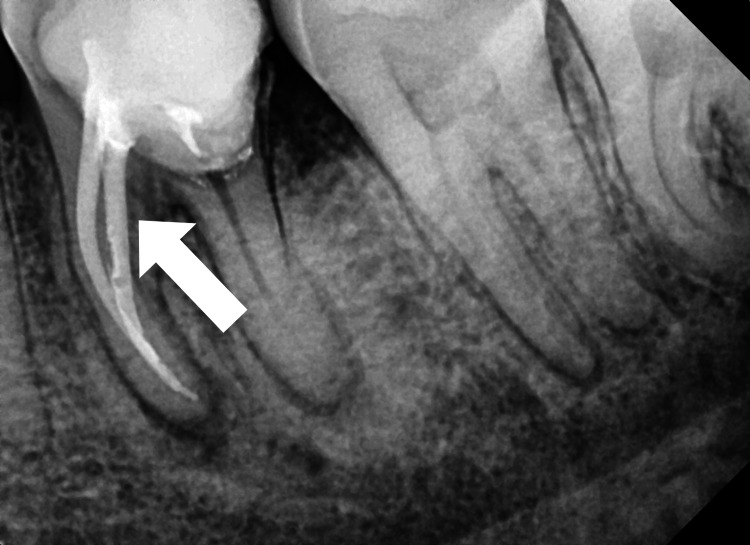
Obturated mesial root of tooth 36 along with final post-endodontic restoration.

The two canals in the mesial root (MB and ML) are obturated with the corresponding master cones and a resin-based sealer (Diadent Dia Proseal Root canal Sealer, South Korea), whereas the distal root canal is left as it is with the tooth number 36. The orifices of the root canals are sealed with Glass ionomer cement, and the bulk of the post-endodontic restoration is built with resin composite (Dentsply Spectrum micro-hybrid composite, Germany) (Figure 3).

**Figure 4:**
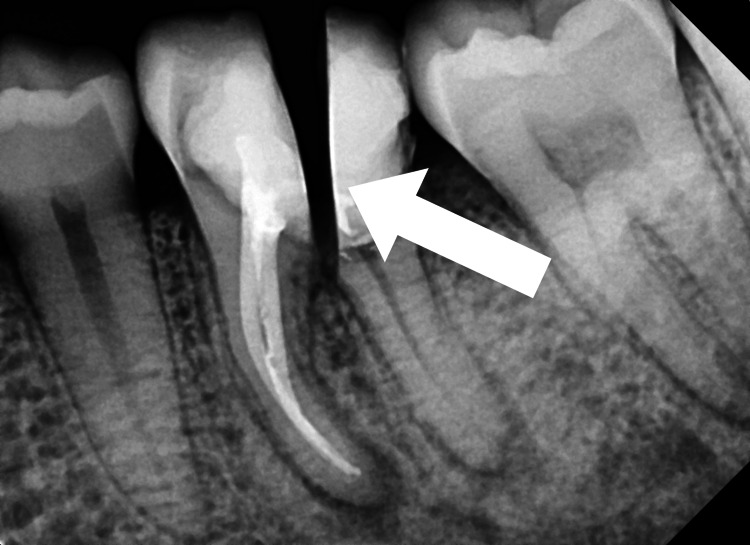
Resection of tooth number 36 using the vertical cut technique

During the third visit, a cervicular incision was made from the second premolar to the second molar, and a full-thickness flap was reflected under local anesthesia. The crown was resected using the vertical cut technique. A carbide bur with a tapered shank was utilized to create a vertical cut in the direction of the bifurcation area (Figure 4).

**Figure 5:**
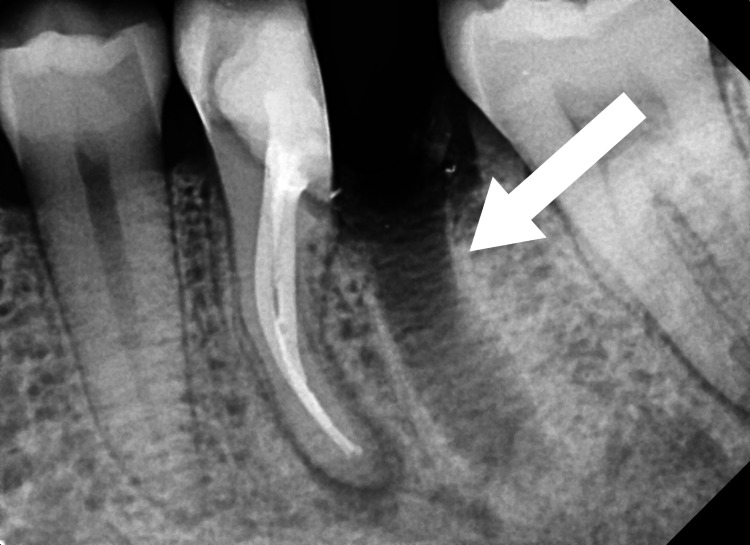
Immediate post-operative radiograph showing the clear extraction socket following the removal of the distal segment of tooth number 36

The distal hemisected portion of tooth number 36 was removed using mandibular premolar forceps. After the sectioning, the clearance of the extraction socket is verified by using a periapical radiograph (Figure 5).

Copious saline irrigation was done in the socket to get rid of the bony chips. Platelet-rich fibrin (PRF) was made with the patient's blood. This autologous PRF was placed in the extracted root socket to facilitate better healing [6]. Following this, suturing was done using a 3-0 Vicryl suture using a figure of 8 technique. The patient was recalled after one week for follow-up.

**Figure 6:**
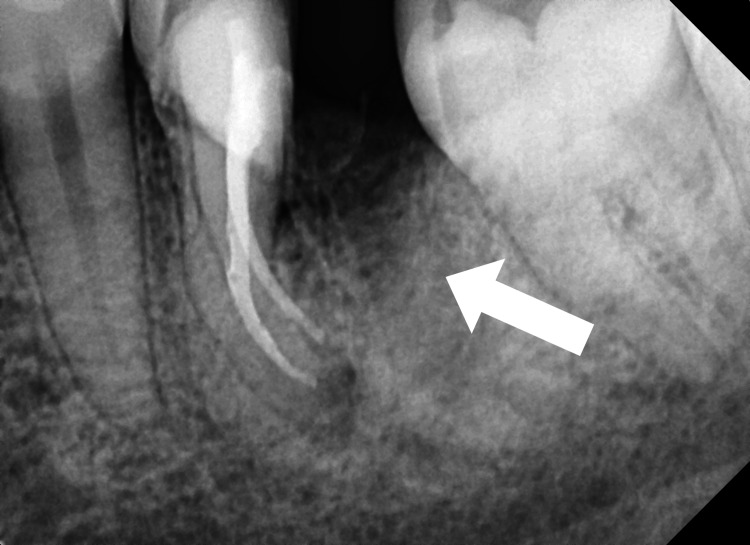
Radiographic follow-up at three months showing the healed extraction socket of the distal root of tooth number 36

The patient was recalled again after three months for follow-up. During this visit, the healing of the extraction socket was analyzed (Figure 6).

**Figure 7:**
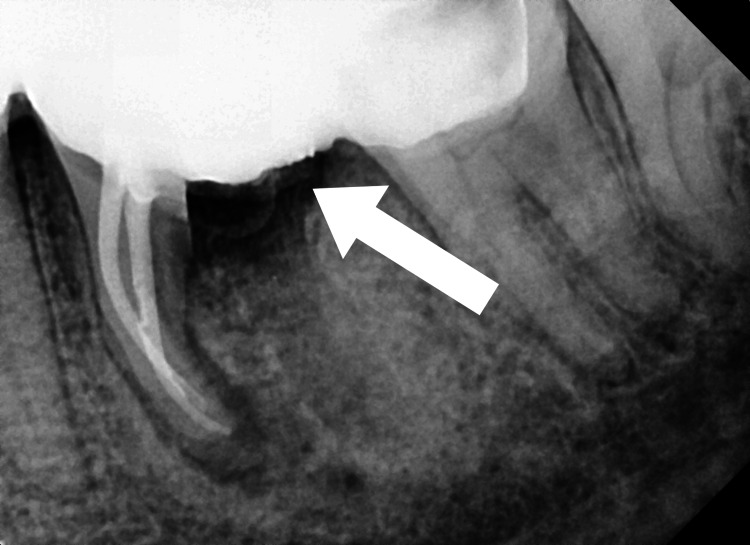
Radiograph showing follow-up at six months for the tooth number 36. The periapical region associated with the mesial root of tooth number 36 displayed a healing lesion. An incomplete resolution of the periapical lesion associated with this root was observed

The patient was then advised to undergo crown prosthesis rehabilitation. At 6-month recall, adequate healing of the extraction socket associated with the distal root of 36 was observed in the radiograph (Figure 7). However, the periapical region associated with the mesial root of tooth number 36 displayed a healing lesion. An incomplete resolution of the periapical lesion associated with this root was observed in the periapical radiograph. This was because of the gross destruction of the surrounding bone that was associated with the condition of endo-perio lesion before the treatment. It is important to note that complete healing of the periapical lesion associated with the mesial root of tooth number 36 is expected to take place within 3-4 years post-treatment.

## Discussion

Hemisection is the sectioning of multi-rooted teeth with their crown portion, with the loss of periodontal attachment, and is performed to retain the original tooth structure and attain the fixed prosthodontic prosthesis [7]. Numerous elements influence the clinician's selection of one treatment strategy over another in the event of a mandibular molar Class III furcation invasion. Three categories can be used to list: (A) A good case selection, diagnostic and treatment planning skills, awareness of therapeutic options, and clinical insight or skill in providing care, which are examples of clinician factors. (B) Patient factors include factors like patient health, the significance of the tooth to the patient, associated expenses, and time constraints. (C) Dental anatomy, mobility, crown-root ratio, degree of attachment loss, inter-arch intra-occlusal relationship, strategic dental value for retention or extraction, and clinical knowledge or proficiency in care delivery.

After the effects of the cause-related therapy have been assessed, the choice of the ultimate course of action should be decided upon. Carnevale proposed a treatment protocol for teeth with furcations [8]. Teeth with furcation involvement have been successfully retained through the hemisected teeth. However, there are a few drawbacks that come with it. Anxiety and pain are possible outcomes, just like with any surgical operation. Caries are more likely to occur on root surfaces that have been ground down at the hemisection or furcation site.

"Root resection" is the combined term for "root amputation" and "hemi-section". The benefit of amputation, hemisection, or bisection, in Newell's opinion [9], is preserving part or all of the tooth. The crown needs to have restorative management performed on it, and the remaining root or roots need to receive endodontic therapy.

Hemisection has the benefit of preserving the tooth and preventing extraction; however, endodontic therapy is required to restore the remaining root or roots. According to Park [10], maintaining good oral hygiene on the part of the patient is essential for the long-term success of hemisection-treated molars.

This clinical report presents a conservative approach to treating an endo-perio lesion. It involves hemisection and prosthetic rehabilitation. When extraction is the recommended course of action, hemisected teeth offer an alternative to extracting the entire tooth while preserving the remaining healthy tooth structure. When done carefully and by the standard protocols, it minimizes the psychological effects of tooth loss on the patient and maintains proprioception, which is essential for the stomatognathic system's normal functioning and prevents damage to the temporomandibular joint.

As long as case selection is done correctly and the restoration, as in this instance, is of an acceptable design about the patient's occlusal and periodontal needs, the prognosis for hemisection is the same as for routine endodontic procedures [11]. Careful case selection and treatment decisions are influenced by a number of factors, such as the experience of the clinician, tooth factors, the tooth's strategic importance, the quality and quantity of the bone, the soft tissue, the accessibility of the operation site, the periodontal status surrounding the remaining root, and, of course, the patient's medical history.

Hemisection may, however, impact the tooth's long-term prognosis if the occlusal surfaces are not appropriately reshaped to create a harmonious contact relationship between the maxillary and mandibular teeth or if the restorative margins are not done correctly. It is important to properly smooth the sharp and rough areas in the furcation region to avoid irritating the gingiva and causing plaque to deposit there. To reduce the forces on the remaining root, which could otherwise fracture, occlusal modification of the remaining tooth is required [12]. Park et al. have proposed that in cases where patients maintain good oral hygiene, hemisection of a tooth may be an option for saving a tooth that would otherwise need to be extracted [10]. Similarly, Saad et al. have determined that if one of the two roots of a mandibular molar is severely damaged and the remaining tooth can function as a healthy abutment, hemisection of the molar is a conservative treatment for extraction [13].

## Conclusions

A precise diagnosis is essential for the successful treatment of endo-perio lesions. The current case report demonstrates the use of a multidisciplinary treatment approach for the management of such kinds of lesions. Factors such as the health of the periodontium, restorative treatment strategy, prosthetic considerations, and patient compliance to maintain oral hygiene play a key role in determining the success of the hemisection procedure. Regular periodontal care at different recall periods is crucial for the long-term survival of the resected tooth. Therefore, hemisection allows the preservation of the remaining healthy natural tooth structure, thus allowing a favorable environment for alveolar bone formation.
